# Editorial: Emerging Markets’ Health and Pharmaceutical Sectors at the Dawn of a Potential Global Financial Crisis of early 2020s

**DOI:** 10.3389/fphar.2022.907612

**Published:** 2022-04-25

**Authors:** Mihajlo Jakovljevic, Demetris Lamnisos, Yuriy Timofeyev, Habib Nawaz Khan, Chhabi Lal Ranabhat, Brian Godman

**Affiliations:** ^1^ Institute of Advanced Manufacturing Technologies, Petersburg Polytechnic University, St.Petersburg, Russia; ^2^ Institute of Comparative Economic Studies, Hosei University, Tokyo, Japan; ^3^ Department of Global Health Economics and Policy, University of Kragujevac, Kragujevac, Serbia; ^4^ Department of Health Sciences, European University Cyprus, Nicosia, Cyprus; ^5^ Graduate School of Business, HSE University, Moscow, Russia; ^6^ University of Science and Technology Bannu, Bannu, Pakistan; ^7^ Department of Health Promotion and Administration, Eastern Kentucky University, Richmond, United States; ^8^ Department of Pharmacoepidemiology, Strathclyde Institute of Pharmacy and Biomedical Sciences, University of Strathclyde, Glasgow, United Kingdom; ^9^ Centre of Medical and Bio-allied Health Sciences Research, Ajman University, Ajman, United Arab Emirates; ^10^ School of Pharmacy, Sefako Makgatho Health Sciences University, Pretoria, South Africa

**Keywords:** emerging markets, BRICS, EM7, economic crisis, recession, pharmaceutical markets, LMICs, big pharma

Essential pharmaceutical innovation in terms of market placement of new chemical entities featuring medicines with novel mechanisms continue to be dominated by Pharmaceutical multinational companies (Sadat et al., 2014). This is gradually changing with the growth of emerging biopharma companies launching their new products rather than being bought over by major Pharma Companies (IQVIA, 2022).

Emerging markets, such as the BRICS (Brazil, Russia, India, China, South Africa) or EM7 (BRIC nations + Mexico, Indonesia, Turkey), serving as the engine of real economic growth worldwide, increasingly shape the demand for medical goods and services. Long-term investment strategies developed by leading industry players emphasize growth opportunities taking place outside wealthy high-income OECD nations, particularly in future decades. BRICs Emerging markets or EM7 are currently at the very epicenter of foreign direct capital investment in most of these strategies and forecasts. Economic slowdown was taking place in Western economies primarily during and after the global macroeconomic crisis 2007-2016. This previous crisis triggered by banktrupcy of brother Lehman has witnessed the endurability and resilience of global emerging markets led by the BRICS, which are findings officially reported by the Brookings Institute and the World Bank. (Raju et al., 2017). After a short recovery of world economy, mostly taking place from 2017–2019, the Corona pandemics attributed global lock-downs, disturbing major supply chains and world trade routes has further stagnated prospects for long lasting market recovery. All of these evolutionary changes reshaped the global macroeconomic landscape and affected the healthcare arena worldwide (Krstic et al., 2020).

“The Silver Tsunami” or population aging, ever growing incidence of chronic and expensive to treat noncommunicable diseases (NCDs) prosperity diseases have jointly led to the increasing gaps in universal insurance coverage (Bartels and Naslund, 2013). This has resulted in a MDG goal to increase universal healthcare as we are seeing in countries such as South Africa (Meyer et al.). Overseeing the Global South perspective has also worsened affordability and equitability of access to medical care for the poor citizen in remote and rural areas unless addressed by donors and other initiatives. There are concerns with healthcare resource allocation and financing strategies in many low- and middle-income countries (LMICs) and sometimes far from being tailored to the local needs (Godman et al., 2019; Godman et al.). At the same time, the share of Developing World nations in the world’s joint health expenditure has grown twice in purchase power parity terms since 1990s (Getzen T. et al., 2016). The necessary health reforms ahead of the LMICs will be shaped by socioeconomic inequalities in medical care access, out-of-pocket spending crossing the threshold of affordability, poor management of supply chains of health commodities, and vulnerabilities against catastrophic household expenditures (Ranabhat et al., 2021). Yet overall growth of living standards in most of the Global South gives us hope and optimism. WHO designated “best buys” interventions such as preventive lifestyle interventions, provision of essential medicines, and spreading of cost-effective basic medical technologies, should all contribute to improved early childhood survival and extended human longevity (Allen et al., 2018). Mainstream features of most Global South nations are third demographic transition, urbanization of mega-scale particularly in Asia, and increased living standards (Jakovljevic et al., 2021).

Developing world countries expose substantial heterogeneity in historical legacy of their healthcare establishments, provision and financing. Epidemiological transition in morbidity and mortality structure is probably the most notable common challenge (Douthit and Astatk, 2016). The burden of infectious diseases, nutritional disorders, and traumatism is gradually being replaced by chronic NCDs. Decreasing working ability, absenteeism, and premature mortality is enhanced by NCDs (Jakovljevic et al., 2019). The national health systems of LMICs face a double burden from these dual long-term trends (Reshetnikov et al., 2019). During and after the COVID-19 Pandemic—these bottle neck inefficiencies of health systems are likely to become even more overstretched (Hodkinson et al., 2020). The macroeconomic global crisis triggered by governmental sanitary measures and closure of borders has impacted on trade and finances for healthcare (Czerny et al., 2021). Years ahead of us will expose an array of crucial vulnerabilities that health systems expose once they are put to the limits of their endurance (Jakovljevic et al., 2020a).

Frontiers in Pharmacology published an original research paper entitled ‘Impact of the low-price medicine policy on medicine supply in China: an interrupted time-series analysis by Zhao et al. The authors showed that low price medicine policy significantly decreased the supply of medicines in total and continuous supply subgroup but there was no effect on intern supply subgroups. Hence, the low-price medicine policy is more useful for severely shortage medicines/drugs (Jakovljevic et al., 2020b).

Subsequently, in 2021, Zhao et al. published another research article entitled “Heterogeneity in Price Elasticity of Medicine Demand in China: Moderate Effect from Economic Incentive and Quality Difference.” It explored that the price elasticity of medicines depends upon the categories of medicines in question; however, there was least elasticity for anti-tumor medicine and the majority of cardiovascular medicine. The authors further found that the absolute value of price elasticity of generic medicine is higher than that of originator medicine in the anti-tumor and CVD therapeutic classes (Mingyue et al., 2021).

There is another research article entitled on “The Impact of Reimbursement Practices on the Pharmaceutical Market for Off-Patent Medicines in Slovakia” in Frontier in Pharmacology authored by Tesar et al. The authors showed that sales of generic medicines were not consistent. In Slovakia, these increased by less than 1%, in Poland and Hungary they decreased by 5%, and in Czechia they decreased by 9% from 2015 to 2020. Likewise, from 2015 to 2020, the sales of biosimilars increased from 0.94 to 2% in Slovakia, from 0.59 to 1.29% in Poland, from 0.72 to 2.23% in Hungary, and from 0.76 to 2.15% in Czechia. So, it is necessary to review the associated policies of drugs supply chain along with associated demand-side measures to assess the rationale for these changes and their implications for the future.

In conclusion we observe dynamic developments at the world pharmaceutical and healthcare markets. Substantial share of this supply and demand is increasingly coming from global emerging markets. Typically, these are the BRICS countries followed by Next Eleven nations. Yet this supply and demand is largely dominated by China, India and South-East Asian ASEAN countries. Profound understanding of this complex and unpredictable landscape is crucial for long term R&D investment strategies. Editors hope that contributions within this Topic have filled certain knowledge gaps (Jakovljevic and Getzen, 2016).
